# Association between magnesium depletion score and atherosclerotic cardiovascular disease: Findings from NHANES 2005 to 2018

**DOI:** 10.1097/MD.0000000000043914

**Published:** 2025-08-15

**Authors:** Tianjiao Liu, Jie Wang, Chenghuan Ren, Ruotong Yu, Changgeng Fu

**Affiliations:** a Department of Xiyuan Hospital, China Academy of Chinese Medical Sciences, Beijing, China; b Department of Graduate School, Beijing University of Chinese Medicine, Beijing, China.

**Keywords:** atherosclerotic cardiovascular disease, cross-sectional study, magnesium depletion score, NHANES

## Abstract

Magnesium deficiency plays a role in the onset and progression of cardiovascular disorders (CVD), and early detection of magnesium deficiency in the body is crucial. Magnesium depletion score (MDS) is a more precise measurement to evaluate the magnesium status of the body. Atherosclerotic cardiovascular disease (ASCVD) is the primary cause of death from CVD, and the correlation between MDS and the prevalence of ASCVD remains unclear. This study aims to analyze the cross-sectional relationship of MDS with ASCVD. This investigation included 26,767 participants from National Health and Nutrition Examination Survey, and 2624 belonged to the ASCVD group. MDS was calculated and separated into 3 cohorts (low, 0–1; medium, 2; high, 3–5). The association between MDS and ASCVD was evaluated using weighted logistic regression, subgroup analysis, and sensitivity analysis. ASCVD prevalence in America was significantly correlated with MDS, according to logistic regression analysis. The prevalence of ASCVD increased by 15% (odds ratio, 1.15; 95% confidence interval, 1.05–1.27) for every unit rise in MDS. Participants with high MDS demonstrated a significantly higher prevalence of ASCVD. This was observed in individuals with high MDS compared to those with low MDS (odds ratio, 1.43; 95% confidence interval, 1.12–1.82), with a significant trend between groups with different levels of MDS (*P* < .001). The results remained robust and consistent in the analysis of subgroups and sensitivity. MDS is an vital risk factor for the prevalence of ASCVD patients.

## 1. Introduction

As a main cause of death worldwide, CVD has an increasing incidence year by year.^[1–3]^ Atherosclerotic cardiovascular disease (ASCVD) is the primary cause of death from CVD,^[4]^ a series of basic diseases with atherosclerosis as the underlying lesion, mainly including coronary heart disease (CHD) and ischemic stroke.^[5]^ Although the promotion of secondary prevention of CVD, modification of cardiovascular risk factors, and revascularization can alleviate the severe symptoms of atherosclerotic disease, the increasing incidence of obesity and diabetes has adversely affected the epidemiological trend of ASCVD, so that the burden of CVD continues to increase.^[6,7]^ The prevention and treatment of ASCVD remain a global problem.

Magnesium is a crucial nutrient for preventing cardiovascular diseases, playing a vital role in regulating vascular smooth muscle, maintaining normal cardiac conduction, modulating vascular endothelial function, anti-inflammation, anti-thrombosis, etc.^[8,9]^ Previous investigations have revealed that magnesium deficiency is associated with the development of atherosclerosis, hypertension, and stroke,^[10,11]^ and the mechanism may be that magnesium deficiency activates the neuroendocrine pathway, causing an inflammatory response with immunological and oxidative stress that affects lipoprotein metabolism and vascular endothelial function.^[12,13]^ According to a survey, magnesium deficiency is very common in the United States.^[14]^ Although, low serum magnesium is considered to be a predictor of cardiovascular and all-cause death,^[15]^ but it cannot timely reflect whether the body has magnesium deficiency.^[16–18]^ The magnesium tolerance test is considered a gold standard to measure the body’s magnesium status, its widespread usage in clinical practice and research is hindered by its complexity.^[14,19]^ It is necessary to use a new and more accurate calculation method for judging magnesium deficiency and use it to intervene in magnesium deficiency early. Magnesium depletion score (MDS) is an ideal index to evaluate the magnesium status of the human body, which fully takes into account the reabsorption of magnesium by the kidney and is simple to calculate, making it easy to be widely used in clinical practice.^[17]^ The higher the MDS, the more serious the magnesium deficiency.^[20]^ According to earlier research, MDS and the prevalence of cardiovascular disease had a positive correlation, and a strong correlation exists between high MDS and an elevated risk of all-cause mortality as well as CVD.^[21]^ But, as far as we are aware, no research has yet investigated the cross-sectional relationship between MDS and ASCVD.

The purpose of this study was to investigate the cross-sectional relationship between magnesium status (as assessed by MDS) and the prevalence of ASCVD in US adults. The tertiary prevention of cardiovascular disease would benefit from this research.

## 2. Methods

### 2.1. Date acquisition

**Figure 1.**
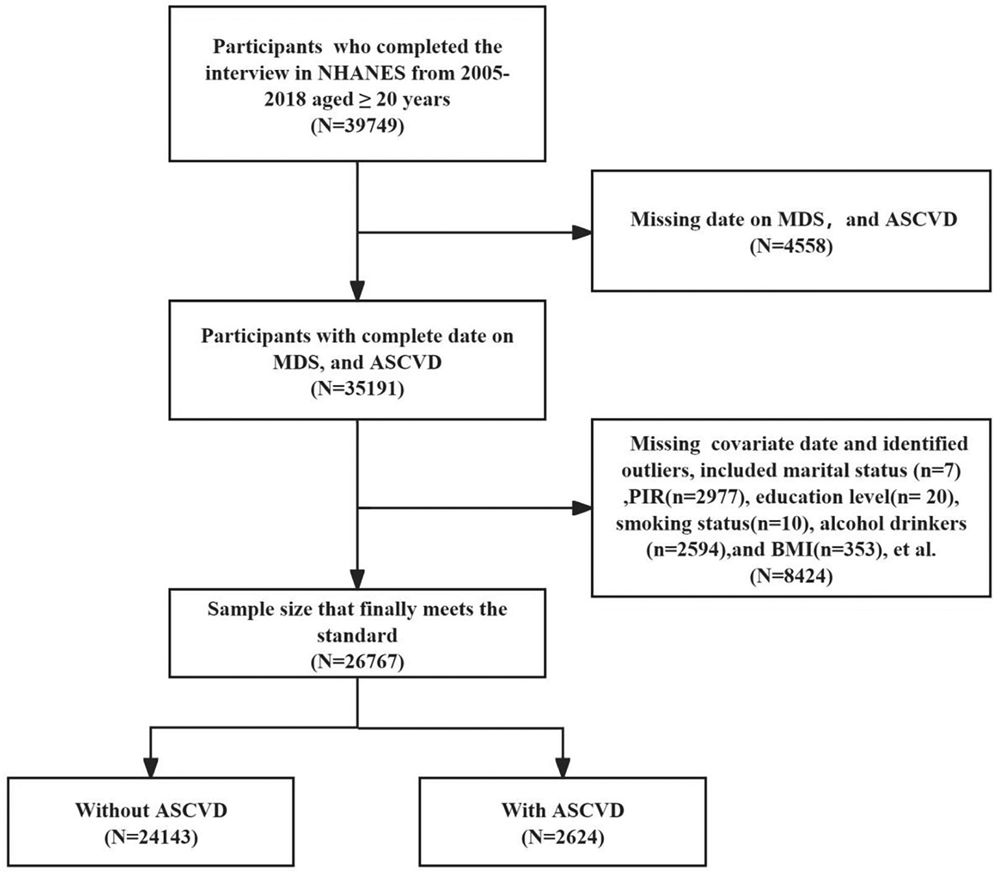
Flowchart depicting the participants’ selection.

The National Center for Health Statistics (NCHS) uses the multistage, stratified design of the National Health and Nutrition Examination Survey (NHANES) to collect comprehensive, nationally representative data about the physical health and nutrition of Americans. American individuals 20 years of age and older who took part in NHANES between 2005 and 2018 made up the participants of the cross-sectional study. Participants in the study were not permitted if they met any of the following exclusion criteria: (1) missing date on MDS (n = 4558) and ASCVD (n = 6); (2) missing covariate data and identified outliers (n = 8424): marital status (n = 7), household poverty-to-income ratio (PIR) (n = 2977), education level (n = 20), smoking status (n = 10), alcohol drinkers (n = 2594), body mass index (BMI) (n = 353), high-density cholesterol (HDL-C) (n = 1267), diabetes (n = 528), hypertension (n = 2), and a family history of heart attack (n = 666). Twenty-six thousand seven hundred sixty-seven people were taken into consideration after the participants were eliminated (Fig. 1). The Institutional Review Board at NCHS granted ethical clearance prior to the survey, and each participant signed the required paperwork to confirm their informed permission.

### 2.2. MDS assessment

The 4 following scores add up to the MDS: (1) the current use of proton pump inhibitors (PPI) was assigned a score of 1; (3) the estimated glomerular filtrate (eGFR) of diuretics was 60 mL/min/1.73 m^2^ ≤ 1; (2) the eGFR was assigned a score of 1 if it was <90 mL/min/1.73 m^2^, or 2 if it was <60 mL/min/1.73 m^2^; (4) heavy drinking (>1 drink/day for women, >2 drinks/day for men) was assigned a score of 1.^[20]^ To compute eGFR, we utilized the Chronic Kidney Disease Epidemiology Collaboration.^[22]^ For ease of analysis and application, MDS was classified into 3 groups: low, 0 to 1; medium, 2; high, 3 to 5.^[23]^

### 2.3. Definition of ASCVD

The Guidelines for the Treatment of Blood Cholesterol to Reduce ASCVD Risk in Adults defined ASCVD as a disorder that could cause angina, a heart attack, a stroke, or at least one kind of CHD. One strict criterion was having a history of heart attacks or strokes. The questionnaire on the CDC-NCHS website contains all of the aforementioned data.^[24,25]^

### 2.4. Assessment of covariates

Covariates were rationally selected to minimize confounding bias in the study through previous studies and in conjunction with the clinic. These covariates included sociodemographic characteristics (age, sex, marital status, race, education status, PIR, and BMI), living behaviors (smoking and drinking status), dietary data (magnesium intake, total energy), comorbidities (hypertension, diabetes), laboratory information HCL-C and total cholesterol, and family history of heart disease. Referring to the Standards of Medical Care in Diabetes, diabetes was identified by self-report, the use of diabetes drugs, or hemoglobin A1c levels ≥ 6.5%.^[26]^ Antihypertensive drug use, self-reported diagnosis, or systolic/diastolic blood pressure ≥ 140/90 mm Hg were used to diagnose hypertension. Data on family history of heart disease were obtained from self-reports asking participants if they had any near relatives who had received a diagnosis of angina or a heart attack from a medical expert before turning 50.

### 2.5. Statistical analyses

To derive a nationally representative analysis and consider the complexity of the NHANES survey design,^[27,28]^ we weighted the data using MEC weights. For pooled analyses of NHANES data from 2005 to 2018, sampling weights were calculated as follows:1/7 × WTMEC2YR. Participant characteristics were described as the mean (standard error), median interquartile spacing, or percentage. In this case, continuous variables normally distributed or approximately normally distributed were described by mean (standard error), skewed variables by median and interquartile spacing, and categorical variables were described by percentage. To ensure the accuracy of the results, we considered confounding factors.

To investigate the relationship between MDS and ASCVD, 3 independent multivariate weighted logistic regression models were created. There were no adjusted covariates in model 1. When creating model 2, age, sex, race, marital status, PIR, and education level were all taken into account. Model 3 was built on Model 2 and additionally adjusted for BMI, smoking status, HDL-C, total cholesterol, average energy intake, diabetes, hypertension, and a family history of heart attack. Additionally, we examined the potential connection between the MDS components (diuretic use, PPI use, drinking, eGFR) and ASCVD.

To investigate whether other factors may have an impact on the relationship between MDS and ASCVD, we performed subgroup analyses that took into account the participant’s age, sex, BMI, smoking status, diabetes status, and family history of heart disease, and examined whether there were any possible interactions between these subgroup characteristics. In addition, to examine how strong this correlation is, we used the multiple imputation method to deal with missing data and then performed sensitivity analyses, again examining the association between MDS and ASCVD when MDS was used as continuous data and under different MDS levels (low, moderate, and high).

All data were processed with NHANES analytic guidelines. R, version 4.1.1, and the statistics program from the Free Software Foundation, version 1.9.2, were applied to statistical analysis. In all statistical analyses, a bilateral *P*-value threshold of <.05 was considered statistically significant.

## 3. Results

### 3.1. Participant characteristics

The description of the participants is shown in Table 1. Baselines of all patients were analyzed in 2 groups based on whether patients had ASCVD. Twenty-six thousand seven hundred sixty-seven participants were included in the study (13,402 males and 13,365 females). Two thousand six hundred twenty-four belonged to the ASCVD group with a mean age of 66.00 (12.82), of which 59.49% were male and 40.51% were female. In comparison, the non-ASCVD group had 24,143 individuals with a male-to-female ratio of 49.05:50.59 and a mean age of 47.62 (17.09). Compared with non-ASCVD patients, ASCVD patients had a higher age, BMI, MDS, and significantly higher prevalence of hypertension, diabetes, and a family history of heart attack, while their average daily magnesium intake, HDL-C levels, and eGFR levels were lower (all *P *< .05).

**Table 1:** Baseline characteristics of participants.

Variables	Total (n = 26,767)	Non-ASCVD (n = 24143)	ASCVD (n = 2624)	*P*-value
Age (years)	49.43 (17.59)	47.62 (17.09)	66.00 (12.82)	<.0001
Sex (%)				<.0001
Male	13,402 (50.07)	11,841 (49.05)	1561 (59.49)	
Female	13,365 (49.93)	12,302 (50.95)	1063 (40.51)	
Race (%)				<.0001
Mexican American	12,149 (45.39)	10,646 (44.10)	10,646 (44.10)	
Non-Hispanic White	5452 (20.37)	4925 (20.40)	527 (20.08)	
Non-Hispanic Black	4125 (15.41)	3867 (16.02)	258 (9.83)	
Other race	5041 (18.83)	4705 (19.49)	336 (12.80)	
Marital status (%)				<.0001
Never	4732 (17.68)	4559 (18.88)	173 (6.59)	
Divorced/separated/widowed	5892 (22.01)	4928 (20.41)	964 (36.74)	
Living with partner	5892 (22.01)	14,656(60.70)	1487 (56.67)	
PIR (median [IQR])	2.19 [1.15,4.18]	2.26 [1.16,4.28]	1.74 [1.03,3.22]	<.0001
Education level (%)				<.0001
<9th grade	6136 (22.92)	5320 (22.04)	816 (31.10)	
High school grade/GED	6162 (23.02)	5478 (22.69)	684 (26.07)	
College graduate or above	14,469 (54.06)	13,345 (55.27)	1124 (42.84)	
BMI (kg/m^2^)	9.27 (6.95)	29.15 (6.93)	30.39 (7.06)	<.0001
Smoking status (%)				<.0001
Never	14,612(54.59)	13,625(56.43)	987 (37.61)	
Former	6636 (24.79)	5576 (23.10)	1060 (40.40)	
Now	5519 (20.62)	4942 (20.47)	577 (21.99)	
Alcohol intake (%)				<.0001
Never	3575 (13.36)	3237 (13.41)	338 (12.88)	
Former	4350 (16.25)	3546 (14.69)	804 (30.64)	
Mild	9139 (34.14)	8196 (33.95)	943 (35.94)	
Moderate	4225 (15.78)	3976 (16.47)	249 (9.49)	
Heavy	5478 (20.47)	5188 (21.49)	290 (11.05)	
HDL-C (mg/dL)	52.88 (16.04)	53.18 (16.029)	50.09 (15.85)	<.0001
TC (mg/dL)	5.00 (1.08)	5.03 (1.06)	4.68 (1.16)	<.0001
Average energy intake (kcal)	2124.83 (998.33)	2152.22 (1005.51)	1872.74 (891.13)	<.0001
Average magnesium intake (mg/day) (median [IQR])	268 [195,366]	271 [198,369]	245 [173,330]	<.0001
eGFR (mL/min)	93.62 (23.49)	95.81 (22.38)	73.44 (23.89)	<.0001
Diabetes (%)				<.0001
No	21,844 (81.61)	20,324 (84.18)	1520 (57.93)	
Yes	4923 (18.39)	3819 (15.82)	1104 (42.07)	
Hypertension (%)				<.0001
No	15,435 (57.66)	14,859 (61.55)	576 (21.95)	
Yes	11,332 (42.34)	9284 (38.45)	2048 (78.05)	
A family history of heart attack (%)				<.0001
No	23,284 (86.99)	21,278 (88.13)	2006 (76.45)	
Yes	3483 (13.01)	2865 (11.87)	618 (23.55)	
Diuretic use (%)				<.0001
No	11,245 (42.01)	11,050 (45.77)	195 (7.43)	
Other	11,792 (44.05)	10,289 (42.62)	1503 (57.28)	
Yes	3730 (13.94)	2804 (11.61)	926 (35.29)	
PPI use (%)				<.0001
No	24,311 (90.82)	22,252 (92.17)	2059 (78.47)	
Yes	2456 (9.18)	1891 (7.83)	565 (21.53)	
MDS (%)				<.0001
Low (0–1 point)	20,719 (77.41)	19,529 (80.89)	1190 (45.35)	
Middle (2 points)	4087 (15.27)	3288 (13.62)	799 (30.45)	
High (3–5 points)	1961 (7.33)	1326 (5.49)	635 (24.20)	

ASCVD = atherosclerotic cardiovascular disease, BMI = body mass index, eGFR = estimated glomerular filtrate, GED = General Educational Development, HDL-C = high-density cholesterol, IQR = interquartile range, MDS = magnesium depletion score, NHANES = National Health and Nutrition Examination Survey, PIR = household poverty-to-income ratio, PPI = proton pump inhibitors, TC = total cholesterol.

### 3.2. Association in MDS and ASCVD

Table 2 shows the weighted multivariate logistic regression analysis results. When MDS was analyzed as a continuous variable, in the model without confounding factor adjustment, MDS and ASCVD showed a positive correlation (model 1: odds ratio [OR], 2.24; 95% confidence interval [95% CI] 2.12–2.37). In models 2 and 3, the positive connection persisted even after confounding factors were taken into account. Model 3 (OR, 1.15; 95% CI 1.05–1.27) indicated a 15% increase in the probability of ASCVD incidence for every unit increase in MDS. According to the analysis’s findings at various MDS levels, those with high MDS levels are substantially more likely to develop ASCVD than people with low MDS levels in all 3 models. For the component items of MDS, diuretic use, PPI use, and renal insufficiency (eGFR < 90 mL/min/1.73 m^2^) were positively associated with a high risk of ASCVD, whereas heavy alcohol use was negatively associated before model adjustment, and no statistical difference was observed after model adjustment (Table 2).

**Table 2:** Weighted multivariate logistic regression models of MDS with ASCVD.

	Model 1		Model 2		Model 3	
	OR (95% CI)	*P*-value	OR (95% CI)	*P*-value	OR (95% CI)	*P*-value
**MDS**						
**Continuous**	2.24 (2.12,2.37)	<.001	1.38 (1.28,1.47)	<.001	1.15 (1.05,1.27)	.004
**Categories**						
Low 0–1 points	Reference		Reference		Reference	
Middle 2 points	3.66 (3.19,4.19)	<.001	1.51 (1.30,1.77)	<.001	1.19 (1.00,1.43)	<.001
High 3–5 points	8.29 (7.04,9.77)	<.001	2.35 (1.95,2.82)	<.001	1.43 (1.12,1.82)	<.001
*P* for trend	*P* < .001		*P* < .001		*P* < .001	
**Diuretic use**	20.72 (17.03,25.21)	<.001	7.26 (5.82,9.05)	<.001	3.87 (3.02,4.97)	<.001
**PPI use**	3.28 (2.80,3.83)	<.001	1.74 (1.46,2.08)	<.001	1.36 (1.13,1.63)	<.001
**Heavy drinking**	0.52 (0.42,0.63)	<.001	1.40 (1.12,1.74)	.003	1.18 (0.92,1.50)	.189
**eGFR**						
0 points	Reference		Reference		Reference	
1 points	3.6 (3.15,4.12)	<.001	1.24 (1.06,1.46)	<.001	1.27 (1.08,1.50)	.004
2 points	12.10 (10.29,14.22)	<.001	1.65 (1.25,2.47)	<.001	1.76 (1.44,2.14)	<.001
*P* for trend	*P* < .001		*P* < .001		*P* < .001	

Model 1: no covariates were adjusted; model 2: adjusted for age, sex, race, marital status, PIR, and education; model 3: adjusted for age, sex, race, marital status, PIR, education level, BMI, smoking status, HDL-C, TC, average energy intake, diabetes, hypertension, and a family history of heart attack.

95% CI = 95% confidence interval, ASCVD = atherosclerotic cardiovascular disease, eGFR = estimated glomerular filtrate, HDL-C = high-density cholesterol, MDS = magnesium depletion score, OR = odds ratio, PIR = household poverty-to-income ratio, PPI = proton pump inhibitors, TC = total cholesterol.

### 3.3. Subgroup analysis

**Figure 2.**
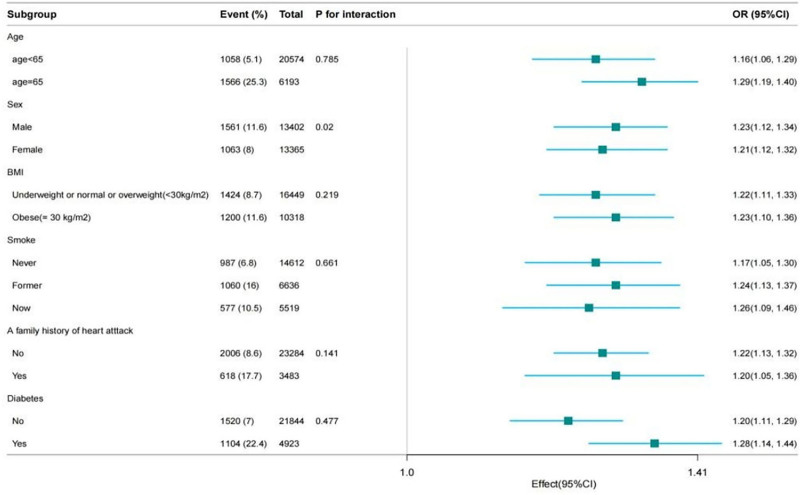
Forest plot of the association between MDS and ASCVD. ASCVD = atherosclerotic cardiovascular disease, MDS = magnesium depletion score.

Strong associations in MDS and ASCVD remained in subgroup analysis. Participants 65 years of age and older had a 29% increased in the probability of developing ASCVD for every unit rise in MDS (OR, 1.29; 95% CI 1.19–1.40). This correlation was also present in the participants whose age under 65 (OR, 1.16; 95% CI 1.06–1.29). The association still remained consistent in the other subgroups (BMI, smoking status, whether have diabetes, etc). In addition, we found that the relationship between MDS and ASCVD was stronger in male (*P* interaction = .020) participants than in female participants (Table 3, Fig. 2).

**Table 3:** Subgroup analysis for the association between MDS and ASCVD.

	OR (95% CI)	*P*-value	*P* for interaction
Age			.785
<65	1.16 (1.06–1.29)	<.001	
≥65	1.29 (1.19–1.40)	<.001	
Sex			.020
Male	1.23 (1.12,1.34)	<.001	
Female	1.21 (1.12,1.32)	<.001	
BMI			.219
Underweight or normal or overweight (< 30kg/m^2^)	1.22 (1.11,1.33)	<.001	
Obese (≥30 kg/m^2^)	1.23 (1.10,1.36)	<.001	
Smoking status			.611
Never	1.17 (1.05,1.30)	.001	
Former	1.24 (1.13,1.37)	<.001	
Now	1.26 (1.09,1.46)	.003	
A family history of heart attack			.141
No	1.22 (1.13,1.32)	<.001	
Yes	1.20 (1.05,1.36)	.01	
Diabetes			.477
No	1.20 (1.11,1.29)	<.001	
Yes	1.28 (1.14,1.44)	<.001	

95% CI = 95% confidence interval, ASCVD = atherosclerotic cardiovascular disease, BMI = body mass index, MDS = magnesium depletion score, OR = odds ratio.

### 3.4. Sensitivity analysis of MDS and ASCVD

Referring to the previous logistic analysis model adjustment, 3 models were established for multivariate logistic regression analysis on the data after multiple interpolation, and the results of all 3 model analyses confirmed that: when MDS was analyzed as continuous variable data, MDS was revealed to be positively associated with ASCVD (OR, 1.25; 95% CI, 1.19–1.30); when MDS was categorical data, this relationship was still consistent. The ASCVD prevalence increases with MDS, with an OR of 1.68 (95% CI, 1.48–1.91) in the high MDS group (Table 4). These results demonstrate the robustness of the study.

**Table 4:** Sensitive analysis of the association between MDS and ASCVD after multiple interpolation.

MDS	OR (95% CI), *P*-value		
	Model 1	Model 2	Model 3
Continuous	2.22 (2.15,2.29) < .001	1.43 (1.37,1.49) < .001	1.25 (1.19,1.30) < .004
Categories			
Low MDS (0–1 points)	Reference	Reference	Reference
Middle MDS (2 points)	3.95 (3.63,4.29) < .001	1.65 (1.50,1.82) < .001	1.36 (1.22,1.51) < .001
High MDS (3–5 points)	7.66 (6.95,8.44) < .001	2.46 (2.19,2.76) < .001	1.68 (1.48,1.91) < .001
*P* for trend	*P* < .001	*P* < .001	*P* < .001

Model 1: no covariates were adjusted; model 2: adjusted for age, sex, race, marital status, PIR, and education; model 3: adjusted for age, sex, race, marital status, PIR, education level, BMI, smoking status, HDL-C, TC, average energy intake, diabetes, hypertension, and a family history of heart attack.

95% CI = 95% confidence interval, ASCVD = atherosclerotic cardiovascular disease, HDL-C = high-density cholesterol, MDS = magnesium depletion score, OR = odds ratio, TC = total cholesterol.

## 4. Discussion

We investigated a representative national sample of American adults in order to show a strong association between elevated MDS and an increased likelihood of prevalence. Interestingly, the relationship between MDS and ASCVD remained significant (*P* < .05) even after conflating factors were taken into account. Additionally, subgroup analyses revealed differences in the associations between the 2 across demographic groups (age, sex, BMI, smoking status, etc). Sensitivity analysis then confirmed the stability of these results.

Magnesium deficiency is closely associated with the development of cardiovascular disease. Earlier epidemiological studies showed that magnesium-deficient drinking water increased the risk of coronary atherosclerosis,^[29,30]^ and higher dietary magnesium intake was associated with a reduced risk of CHD.^[31,32]^ A meta-analysis by NHJ et al, in vitro and animal studies^[33]^ showed that magnesium deficiency was associated with heart failure, arrhythmia, arterial calcification, and endothelial dysfunction, and that magnesium supplementation improved the above conditions and improved clinical cardiovascular outcomes, and suggested that the mechanisms involved may be related to the involvement of magnesium in the establishment and maintenance of the transmembrane potential and the modulation of various ion channels,^[34,35]^ in counteracting vascular calcification,^[36]^ in balancing the endothelial and smooth muscle function and thus regulating vascular tone,^[37,38]^ and in lowering low-density lipoprotein cholesterol and triglycerides.^[39]^ In animal experiments, magnesium supplementation could reduce cholesterol deposition in the vascular wall of experimental animals and had anti-atherosclerotic effects in APOE mice fed a low-fat regular chow diet.^[40,41]^ Clinical studies have found that magnesium supplementation can improve blood pressure, endothelial function and preclinical atherosclerosis in female hypertensive patients taking oral thiazide antihypertensive drugs.^[42]^

Most previous related studies have focused on the impact of dietary magnesium, serum magnesium, and circulating magnesium on cardiovascular diseases, including ASCVD, while neglecting the body’s magnesium status, that is, whether the body is deficient in magnesium. Moreover, it has been confirmed that assessing magnesium status solely based on serum magnesium is inaccurate. In some investigations, it was also found that there is no substantial correlation between serum magnesium and magnesium intake.^[43–45]^ Khan et al^[46]^ also believe that serum magnesium cannot be used as a predictor of CVD incidence. MDS is positively correlated with the severity of magnesium deficiency in the body and takes into account the factors affecting magnesium reabsorption, thus being able to accurately assess the body’s magnesium status.^[47]^ Our study provides evidence that MDS is associated with the prevalence of ASCVD.

The probable mechanisms by which magnesium deficiency leads to the increased prevalence of ASCVD are roughly as follows: 1. magnesium participates in regulating the exchange of K+ and protons H+; intracellular magnesium deficiency causes an increase of calcium and sodium ions, arterial spasm, increased catecholamine release, and the deposition of fatty acids and blood lipids.^[48,49]^ 2. Magnesium deficiency is linked to various episodes of the metabolic syndrome, including diabetes, obesity, and hypertension,^[47,50]^ and these all lead to the susceptibility of various CVDs, including ASCVD. Related studies have shown a connection between magnesium deficiency and increased serum levels of inflammatory markers and induce an inflammatory response through neuroendocrine activation, leading to endothelial dysfunction, abnormal lipid metabolism, and thrombosis.^[11,51]^

Our study assessed magnesium deficiency using MDS and explored the relationship between magnesium insufficiency and ASCVD prevalence, which offers a new perspective for the study of the correlation between magnesium and ASCVD. However, our investigation is not without limitations. First, the methodology of the cross-sectional study makes it challenging to establish a causative link between MDS and the incidence and development of ASCVD. Future research using different study methods (such as longitudinal cohort studies) will be necessary to further investigate both the causal association and potential processes. Second, even after adjusting for a great deal of confounders in the analyses, we were unable to rule out bias due to residual confounding. Therefore, in addition to the sensitivity analyses used in this article to assess the potential effect of confounding factors, subsequent studies could quantify these factors with more detailed baseline data collection and use propensity score matching or stratified analyses to balance between-group differences to further improve the reliability of the findings. Furthermore, there is no data on serum magnesium in the NHANES data, thus this section is not covered in the article, although it is worth noting that the current study of MDS is based on static indications. It is proposed that the follow-up study of magnesium insufficiency and cardiovascular disease should combine MDS with serum magnesium, magnesium tolerance test, and other dynamic markers to increase the study’s sensitivity and accuracy. Finally, the study participants were U.S. adults (Table 1 shows the races included), so subsequent studies should add data from different countries and ethnic groups to improve the evidence-based evidence chain of MDS through multicenter collaboration and longitudinal studies and improve the generalizability of the findings.

## 5. Conclusions

MDS was positively associated with the prevalence of ASCVD. Subjects with greater MDS had a noticeably increased prevalence of ASCVD than did those with lower MDS.

## Acknowledgments

Sincere gratitude to all of the NHANES participants from 2005 to 2018 and all staff of the National Center for Health.

## Author contributions

**Conceptualization:** Tianjiao Liu, Jie Wang.

**Data curation:** Jie Wang, Chenghuan Ren, Ruotong Yu.

**Investigation:** Tianjiao Liu.

**Methodology:** Tianjiao Liu, Jie Wang.

**Resources:** Changgeng Fu.

**Software:** Tianjiao Liu, Jie Wang.

**Supervision:** Changgeng Fu.

**Validation:** Tianjiao Liu, Chenghuan Ren, Ruotong Yu.

**Writing – original draft:** Tianjiao Liu.
